# Feasibility study of using one-tenth mSv radiation dose in young children chest CT with 80 kVp and model-based iterative reconstruction

**DOI:** 10.1038/s41598-019-48946-z

**Published:** 2019-08-28

**Authors:** Jihang Sun, Qifeng Zhang, Di Hu, Yun Shen, Haiming Yang, Chenghao Chen, Zuofu Zhou, Yun Peng

**Affiliations:** 1 Department of Radiology, Beijing Children’s Hospital, Capital Medical University, National Center for Children’s Health, No.56, Nanlishi Road, Xicheng District, Beijing, 100045 China; 2 Department of Radiology, Tokyo Women’s Medical University &Medical Center East, Tokyo, 116-8567 Japan; 3 Respiratory Department, Beijing Children’s Hospital, Capital Medical University, National Center for Children’s Health, No.56, Nanlishi Road, Xicheng District, Beijing, 100045 China; 4 Department of Thoracic surgery, Beijing Children’s Hospital, Capital Medical University, National Center for Children’s Health, No.56, Nanlishi Road, Xicheng District, Beijing, 100045 China; 5 Department of radiology, Fujian Provincial Maternity and Children’s Hospital, affiliated hospital of Fujian Medical University, No.18 Daoshan Road, Gulou District, Fujian, 350000 China

**Keywords:** Respiratory tract diseases, Physical examination, Paediatrics, Paediatric research

## Abstract

CT has become a routine imaging modality based on its excellent ability of displaying lung structures and diseases. But, how to reduce radiation dose of routine CT examination is a concern for radiologists. Our study aimed to evaluate the feasibility of using 80kVp and a model-based iterative reconstruction (MBIR) algorithm to achieve one-tenth mSv dose chest CT in infants and young children. Thirty-two cases (study group, average age 1.71 ± 1.01 years) underwent non-contrast chest CT examination at low dose with 80 kV, 4mAs and was reconstructed with MBIR (LD-MBIR) and the standard adaptive statistical iterative reconstruction (ASIR) algorithm (LD-ASIR); another group (control group) of 32 children underwent routine-dose chest CT with 100 kV and was reconstructed with ASIR only (RD-ASIR). The subjective and objective image quality of the three groups were measured and statistically compared. The radiation dose for the low dose scan was 0.09 ± 0.02 mSv, 6% of the routine dose. All LD-MBIR images were diagnostically acceptable. Compared with the RD-ASIR images, the LD-MBIR images were similar in noise in the left ventricle, muscles, lung field, on-par in displaying large airways, lung lucency and mediastinum, but were inferior in displaying lung marking, small airways and mediastinum. Thus, MBIR images with low dose in pediatric chest CT can be used in the diagnosis for lung field and air way disorders in infants and young children.

## Introduction

Lung diseases such as pneumonia, cystic fibrosis as well as interstitial lung disease can be found at any age in life. For instance, in Beijing, China, the mortality rate of pneumonia is the 5^th^ in the causes of death for children under 5 years of age (7.42%) in 2015^1^. It is believed that lung diseases in infancy and young children not only link to lung development and lung injury, but also represents disorders not seen in adult^2^. As these events occur early in life and are usually life-threatening, it is critical to develop techniques for early diagnosis and monitoring lung structural changes in infants and young children.

CT has become a routine imaging modality based on its excellent ability of displaying lung structures^3^ and diseases. But, how to reduce radiation dose of routine CT examination is a concern by radiologists. This is especially important for children, since children in the growth and development period are extremely sensitive to radiation damages, and these damages can cumulate throughout the whole life. In recent years, iterative reconstruction (IR) algorithms such as Adaptive Statistical Iterative Reconstruction (ASIR) have been widely used clinically^4–6^. Studies revealed that these IR algorithms can maintain image quality with a radiation dose reduction of 23% to 66%, including chest CT, abdomen CT and head CT, but a certain amount of image noise and artifacts are still present^7^. A full model-based iterative reconstruction algorithm (MBIR, GE Healthcare, USA) has demonstrated the ability to improve image quality at low radiation dose levels in adults^8^ and in children^9–14^. So, we wanted to evaluate the feasibility of using only 0.1 mSv radiation dose in young children chest CT with 80kVp and reconstructed with MBIR.

## Results

### Patients’ characteristics

In total, 32 pediatric patients (23 males, 9 females, average age 1.71 ± 1.01 years) were enrolled in the study group, and 32 children (18 males, 14 females, average age 1.71 ± 1.01 years) with matched age distribution in the control group. The pathologies of all patients are shown in Table 1.

**Table 1:** Pathologies considered of study group and control group.

	Study group	Control group
Lymphoma in mediastinum	0	2
Neurogenic tumor	2	1
Pulmonary inflammation	20	28
Pulmonary congenital cystic disease	4	0
Post-operation of mediastinum or pulmonary	3	0
Normal thymus	3	1
Total	32	32

### Radiation dose

For the study group, the CTDIvol, DLP and effective dose values were 0.06 ± 0.00mGy, 1.34 ± 0.21mGy-cm and 0.09 ± 0.02 mSv, respectively. For the control group, they were 1.38 ± 0.25mGy, 27.62 ± 6.10mGy-cm, and 1.50 ± 0.51 mSv, respectively. 94.00% effective radiation dose reduction was achieved in the low dose study group compared with the routine dose control group. Radiation dose data followed normal distribution, Differences in radiation dose between the two scans were compared using paired t test. there was statistical difference among 2 group (t = 17.40–28.92, all P < 0.05).

### Subjective image quality evaluation

**Figure 1:**
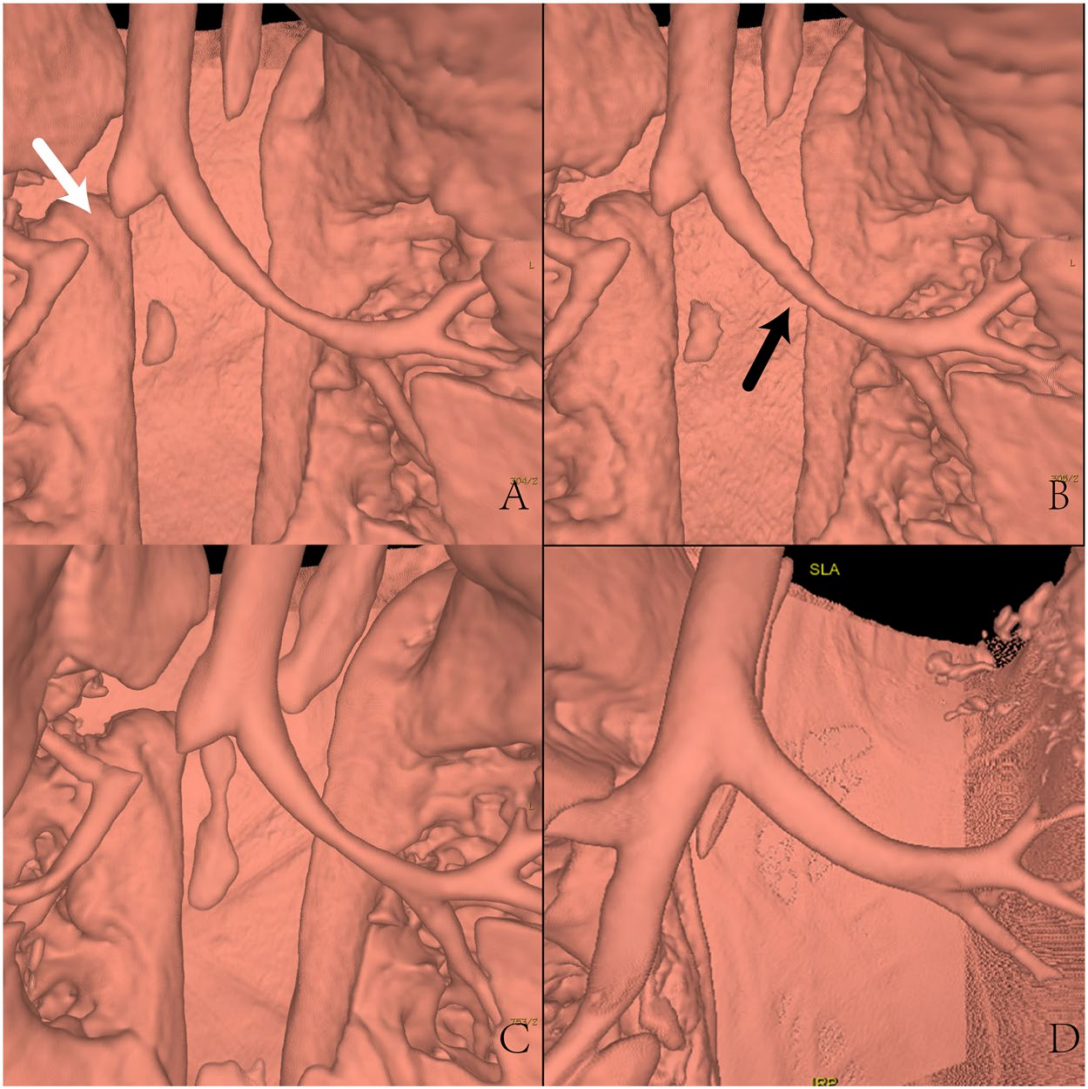
The reconstructed image of airway by Solid Air Technique on AW4.5. (**A**–**C**), a 9 months old boy suffered from pulmonary congenital cystic disease (**A**) Low dose images, radiation dose was 0.10 mSv, reconstructed with MBIR (LD-MBIR). (**B**) Low dose images, reconstructed with ASIR50% (LD-ASIR). (**C**) Routine dose CECT images, radiation dose was 1.64 mSv, reconstructed with ASIR50%. All images displayed the large airways. The airway edge was smooth and sharp on LD-MBIR and RD-ASIR images, less smooth and uneven (black arrow) on the LD-ASIR image. This phenomenon was caused by image noise and was clearer when airway getting thinner. The defects in the right proximal main bronchus marked with white arrow could be completely displayed on all 3 images, indicating small effect of radiation dose on displaying large airways. (D), 9 months old girl with pneumonia, Routine dose non enhanced CT images, radiation dose was 1.64 mSv, reconstructed with ASIR50% (RD-ASIR). Image quality same like (**C**).

**Figure 2:**
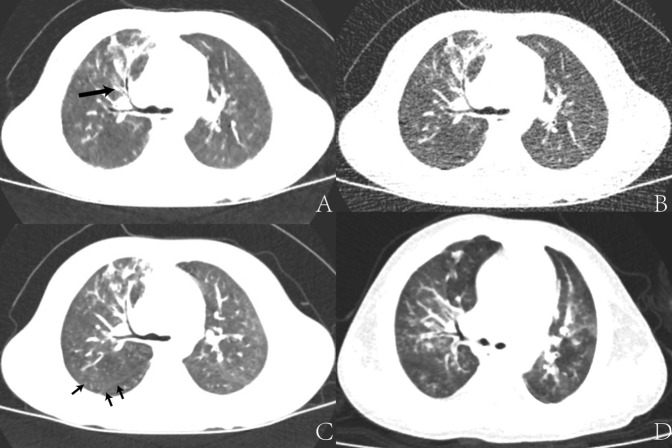
Images of lung window acquired at different dose levels and with different reconstruction algorithms. (**A**–**C**), 3 years old boy with pneumonia. (**A**) Low dose images, radiation dose was 0.08 mSv, reconstructed with MBIR (LD-MBIR). (**B**) Low dose images, reconstructed with ASIR50% (LD-ASIR). (**C**) Routine dose CECT images, radiation dose was 1.34 mSv, reconstructed with ASIR50%. The noise of the LD-MBIR images was similar to that of the Routine dose CECT ASIR images. However, LD-MBIR images were inferior to CECT ASIR images in displaying fine lung markings and pleural shadows (short arrow). The bronchial lumen with LD-MBIR (long arrow) was clearer than LD-ASIR. (**D**) Another 3 years old girl with pneumonia. Routine dose non enhanced CT images, radiation dose was 1.43 mSv, reconstructed with ASIR50% (RD-ASIR). The bronchial lumen was similar to LD-MBIR, but the performance of lung markings was better than LD-MBIR.

**Figure 3:**
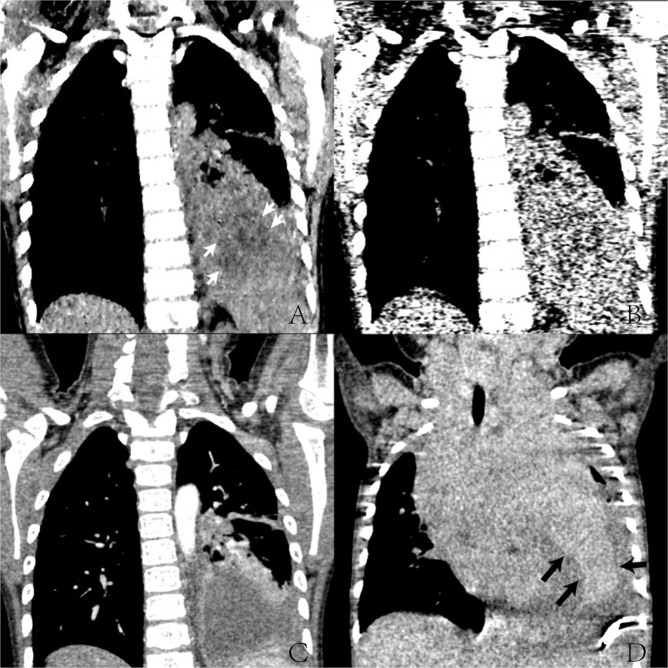
The reconstructed image of coronal section images. (**A**–**C**) 3 years old boy with pneumonia. (**A**) Low dose (0.09 mSv) MBIR image (LD-MBIR), (**B**) Low dose ASIR50% images (LD-ASIR) and (**C**) routine dose CECT ASIR50% image, radiation dose was 1.54 mSv. LD-MBIR images showed a patch of low density in the center of consolidation (white arrow head), but the boundary was blur, which was confirmed by the routine dose CECT image as a necrotic lesion, that LD-ASIR was not able to display it. (**D**) Another 2 years old boy with Lymphoma. Routine dose non enhanced CT images (RD-ASIR), radiation dose was 1.38 mSv, which can display different density well, the boundary was clear (black arrow).

All patient examinations underwent initial quality checks before discharge using the routine dose, contrast-enhanced scan to make sure diagnostic information could be acquired. All subjective scores not accord with normal distribution by statistically analyzing, were compared using Kruskal-Wallis test. The subjective image quality evaluations of the three reconstruction groups had a significant deviation (p < 0.05), and post pairwise comparison analysis results are displayed in Table 2. Generally, the LD-MBIR images were diagnostically acceptable with comparable image quality in displaying large airways (Fig. 1), lung lucency and mediastinum to the RD-ASIR images, but were inferior in displaying small airways and lung markings (Fig. 2). The LD-ASIR images were not able to reach the diagnosis level in all 6 aspects. The subjective scores had significant differences between the 3 image groups (LD-MBIR, LD-ASIR and RD-ASIR) in all 6 aspects. Weighted Kappa analysis showed a high consistency between the two senior radiologists (doctor A and doctor B) for the subjective score with Kappa value of 0.92. The Kappa values decreased to 0.77 between doctor A and doctor C, and 0.84 between doctor B and doctor C (all p < 0.05). A total of 92 lesions were found on MIBR images acquired with low dose CT scanning, including 48 parenchymal infiltration lesions (thickened wall of small airways, atelectasis and necrotizing pneumonia) (Fig. 3), 14 interstitial lesions and shadows of streaks, 19 lesions with changed lung density (obstructive pulmonary emphysema and cystic diseases included), 4 lesions with pleural disease, 5 mediastinal masses and 2 bronchiostenosis. The number of lesions found on the LD-MBIR images was the same as that on the following contrast enhanced images acquired with the routine dose CT. However, LD-ASIR images could only display 87 lesions, five lesions that could not be displayed were all obstructive pulmonary emphysema.

**Table 2:** Pairwise comparison analysis of Kruskal-Wallis test between subjective evaluation of different sequences.

	LD-MBIR		LD-ASIR		RD-ASIR		P-value		
	Median score	percentage	Median score	percentage	Median score	percentage	LD-MBIR and LD-ASIR	LD-MBIR and RD-ASIR	LD-ASIR and RD-ASIR
subjective noise score	3.0	2.0(4.2%); 3.0(66.7%); 4.0(29.2%)	2.0	1.0(10.4%); 2.0(86.5%); 3.0(3.1%)	5.0	5.0(100%)	<0.001	<0.001	<0.001
lung lucency score	3.0	2.0(2.1%); 3.0(65.6%); 4.0(32.3%)	2.0	1.0(29.2%); 2.0(51%); 3.0(19.8%)	5.0	5.0(100%)	<0.001	<0.001	<0.001
lung-markings score	3.0	2.0(16.7%); 3.0(71.9%); 4.0(11.5%)	2.0	1.0(20.8%); 2.0(70.8%); 3.0(8.3%)	5.0	5.0(100%)	<0.001	<0.001	<0.001
small airways score	2.0	2.0(51.0%); 3.0(44.8%); 4.0(4.2%)	2.0	1.0(19.8%); 2.0(72.9%); 3.0(7.3%)	5.00	5.0(100%)	<0.001	<0.001	<0.001
large airway score	4.0	2.0(2.1%); 3.0(29.2%); 4.0(68.2%)	3.0	2.0(45.8%); 3.0(44.8%); 4.0(9.4%)	5.0	5.0(100%)	<0.001	<0.001	<0.001
Mediastinum score	3.0	2.0(6.3%); 3.0(84.4%); 4.0(9.4%)	2.0	1.0(7.3%); 2.0(92.7%)	5.0	4.0(6.3%) 5.0(93.7%)	<0.001	<0.001	<0.001

### Objective image quality

Table 3 lists the objective noise measurement and statistical results. Compared with the LD-ASIR images acquired with the same low dose level, LD-MBIR images displayed dramatically lower objective noise. When compared with the RD-ASIR images, the LD-MBIR images had statistically same noise in left ventricles, muscles and lung field (p = 0.23–0.99), but slightly higher noise in fat (p = 0.04). LD-MBIR images’ noise values were much lower than those of LD-ASIR images, with statistically significant differences (all p < 0.05).

**Table 3:** One-way-ANOVA and pairwise comparison analysis of objective noise measurement among different sequences.

	LD-MBIR	LD-ASIR	RD-ASIR	One-way-Anova	Pairwise comparison analysis			
				F-value	P-value	P-value (LD-MBIR and LD-ASIR)	P-value (LD-MBIR and RD-ASIR)	P-value (LD-ASIR and RD-ASIR)
left ventricles	18.80 ± 2.99	72.25 ± 13.95	16.09 ± 2.55	441.18	<0.001	<0.001	0.31	<0.001
muscles	19.83 ± 2.93	85.11 ± 17.51	16.52 ± 3.41	630.74	<0.001	<0.001	0.23	<0.001
lung field	23.17 ± 4.68	66.64 ± 15.79	23.00 ± 4.27	252.17	<0.001	<0.001	0.99	<0.001
fats	19.48 ± 3.96	69.26 ± 14.68	15.70 ± 3.10	547.73	<0.001	<0.001	<0.001	<0.001

## Discussion

CT scan is needed for children, especially for infants with recurrent pneumonia, refractory wheezing or hemoptysis existed to detect airway lesions. Even enhanced CT is performed to further clarify vascular malformations^15,16^. Since the introduction of IR algorithms, they have been widely applied by different companies^17–20^. Newly developed full model-based iterative reconstruction (MBIR) algorithm is reported to be able to reduce noise more significantly and has been applied in the examination for children’s lungs^12,14^. Previous studies have investigated the feasibilities of acquiring diagnostically acceptable images by combination of low dose and MBIR. However, little report can be found in children, especially combining with low tube voltages. ICPR suggests using low tube voltages for low body weight children with higher contrast, higher noise can be tolerated^21^. So, in our present study, CT scans in children less than 3 years old were performed with the lowest tube voltage at 80 kVp, the minimum current at 10 mA and the fast rotation time at 0.4 s, this scan protocol decreases the CTDIvol to 0.06mGy and the effective radiation dose to less than 0.1 mSv. Images acquired at the low dose scans were reconstructed with two different iterative algorithms and compared with those of state-of-the-art ASIR images at the routine radiation dose in terms of image quality and diagnostic accuracy.

Our study showed that MBIR could reduce noise significantly in young children less than 3 years old. In the aspect of subjective noise, MBIR images showed significantly lower granular noise; the images were more smoothing and exquisite when compared with ASIR50% images acquired with the same low dose. However, the MBIR images were slightly blurred compared with the routine dose ASIR images, yielding lower spatial resolution and reduced subjective image quality scores. Specifically, even though the minute structures (such as lung marking and bronchioles) identified in the low dose MBIR images could be used for localization and quantification, they were inferior to those of the standard-dose ASIR images (Fig. 2). As for large airways, although there were statistic difference between LD-MBIR images and RD- ASIR images (p < 0.05), the subjective score of LD-MBIR images was higher than 4.0, it was able to display the large airways with a good image quality (Fig. 1). While with the same low dose, LD-ASIR images could only display large airways but lost the ability to guarantee the image quality in other aspects, too much image noise could decrease the ability to identify structures with similar density (Fig. 3). The objective noise value of LD-MBIR images was significantly reduced (by 65.23–76.70%) compared to that of LD-ASIR images acquired with the same low radiation dose, and was only slightly higher than that of RD-ASIR images. The LD-MBIR images could display the soft tissue with a good contrast compared to LD-ASIR images. In addition, being a new iterative reconstruction algorithm, the acceptance levels for MBIR in terms of image texture were different among radiologists with different experiences and the levels for senior radiologists with more imaging experience were higher and more consistent.

The Kappa values decreased between junior doctor (Kappa values were 0.77–0.84) to the two senior doctors (Kappa value was 0.92), the difference may be caused by the fact that the senior doctor could not adapt the blurred edges in MBIR images^14^. But the junior doctor had good consistency with senior doctor in density evaluation, like lung lucency and lung marking.

There are several limitations in our study: first, the sample size was small. More cases and detailed information will be collected in future studies; second, in this study we only investigated the feasibility of combining low dose CT and MBIR on the diagnosis of lung disease in infants and young children, considered 80 kV may not be suitable for older children, we strictly limited the inclusion criteria for the study group; Third, in this study, only plain scanning was performed at low dose and reconstructed with MBIR to avoid double exposure to the same patient, the effect of MBIR on enhanced CT was not evaluated. In addition, the scan mode difference between the two dose groups, for example the rotation speeds were different in 2 scanning-protocols, that reduced the motion artifact from breathing in MBIR images, may also have introduced the contrast bias to cause the inferiority of the low dose MBIR images to show small lung markings and airways.

In conclusion, our study suggested that lung field and air way diagnostically acceptable images can be acquired at low radiation dose of 0.09 mSv by combining MBIR and 80 kV tube voltage, which may be used to replace traditional plain chest films.

## Methods

This study was approved by the Ethical Committee of Beijing Children’s Hospital, informed consent had been obtained from all children’s legal guardian before starting CT scans. The study protocols were performed in accordance with the approved guidelines and regulations of our hospital. In this study, consecutive cases of the study group were selected from our hospital between November 7, 2015 and January 15, 2016. All patients were under 3 years old, and the control group was formed by continuously selecting chest non-contrast CT patients from November 7, 2015 to November 16, 2015 with matched age to the study group.

### CT data acquirement

All scans were performed on a multiple-detector row CT scanner (64rows, Discovery CT750 HD, GE Healthcare). Patients were scheduled for non-contrast and contrast-enhanced CT scans. To avoid excessive exposure, the non-contrast scan in the study group used only low dose and without routine dose scan, the contrast-enhanced scan in the same group used the routine dose to ensure clinical diagnosis. Scan parameters for the non-contrast scan in the study group were fixed as following: tube voltage of 80 kV, tube current of 10 mA, Pitch: 1.38, gantry rotation speed of 0.4 s. Raw data obtained from the non-contrast scan (study group) was reconstructed to 0.625 mm thickness images with MBIR (LD-MBIR), and 0.625 mm hybrid images with 50% ASIR and 50% FBP (LD-ASIR). Another group of patients with routine dose non-contrast chest CT was selected as the control group, using the following scan parameters: tube voltage of 100 kV, gantry rotation speed of 0.8 s, tube current was set 100–350 mA and determined by automated tube current modulation (ATCM). The noise index was 11-13HU based on patient age. and the raw data was reconstructed into 0.625 mm hybrid images with 50% ASIR and 50% FBP (RD-ASIR), without any additional filters. All the children were given oral sedation to reduce motion artifacts.

### Subjective image quality assessment

All images were transferred to a GE advanced workstation (AW4.5) and were anonymized for analysis. The image quality assessment was analyzed by two experienced pediatric radiologists (doctor A with 13 years and doctor B with 10 years of experience) and a junior pediatric radiologist (doctor C, with 4 years of experience) independently. Clinical information and scan parameters such as name, age, sex of patients, IR technique was concealed when reviewing images. During the whole assessment, the 3 radiologists were able to adjust image window width and window level freely and then give their own subjective scores independently.

A 5-point scoring system similar to other research^12–14^ was used: 5, excellent, image noise was rare, lung lucency, lung marking and airway was defined well, mediastinal structure’s edge could be distinguished clearly. 4, good, there was some noise in image, lung lucency, lung marking and airway was defined, mediastinal structure’s edge could be distinguished; 3, diagnostically acceptable, the whole image noise was increased, lung lucency, lung marking and airway was affected by image noise, but could be accepted, mediastinal structure’s edge was not clear enough, but could be accepted; 2, image noise was high, whole image quality was less than acceptable, but lung marking’s location and edge of the lesion can be distinguished; and 1, non-diagnostic. Scoring was assessed in 6 aspects including subjective noise, lucency, lung marking, small airways, large airways, and mediastinal condition. The number of lesions on LD-MBIR and LD-ASIR was also recorded; pulmonary segment was used as counting unit for invasive and diffuse pulmonary lesions; unilateral or encapsulated pleural effusion was recorded as 1 lesion.

### Objective noise measurement

After all the subjective image quality evaluations were completed, the two senior doctors draw a regions of interest (ROI) at the maximum cross section of the left ventricle on the AW4.5 workstation, then CT value and standard deviation (SD) were calculated by computer automatically. SD were also measured for the erector spinae, lung field and subcutaneous fat by drawing ROI half of the size of the descending aortic (14–30 mm^2^) cross section on the same slice.

### Radiation dose calculation

volumetric CT dose index (CTDIvol) and dose length product (DLP) were calculated and recorded automatically by scanner after examination. Then we calculated the Effective dose (ED) using the following formula based on the report of ICRP103 for computed tomography^22^:

$${\rm{ED}}={\rm{DLP}}\times {\rm{W}}.$$

W is a conversion factor for the chest area of pediatric patients depended on patient age. We used W values (in mSv-mGy^−1^-cm^−1^) of 0.0739 and 0.0480 at 100 kV and 0.0823 and 0.0525 at 80 kV for the patient groups of 0–12 months and 1–3 years, respectively.

### Statistical analysis

The Kolmogorov-Smirnov test was used to check for normal distribution. CTDIvol and DLP values were compared using paired t test. Since the subjective evaluation data were nonparametric, The Kruskal-Wallis test was used to compare the image quality scores among the 3 algorithms and the Bonferroni analysis was used to compare the difference between the pairwise of LD-MBIR, LD-ASIR and RD-ASIR. The objective evaluation data were normally distributed continuous data, were compared statistically using one-way ANOVA, to account for multiple statistical, a Bonferroni analysis was applied to compare the difference between the pairwise of 3 groups. The inter-observer agreement for the image quality was evaluated by using the Kappa test. All the statistical analysis was performed by using SAS software (version 9.3, SAS, Inc., Cary, NC, USA) with p < 0.05 indicating statistically significant difference.

## Data Availability

The datasets generated during and/or analyzed during the current study are available from the corresponding author upon reasonable request.

## References

[1] Cao H (2017). Trend analysis of mortality rates and causes of death in children under 5 years old in Beijing, China from 1992 to 2015 and forecast of mortality into the future: an entire population-based epidemiological study. BMJ Open..

[2] Deterding RR (2010). Infants and Young Children with Children’s Interstitial Lung Disease. Pediatr Allergy Immunol Pulmonol.

[3] Davis SD (2007). Computed tomography reflects lower airway inflammation and tracks changes in early cystic fibrosis. Am J Respir Crit Care Med.

[4] Katsura M (2012). Model-based iterative reconstruction technique for radiation dose reduction in chest CT: comparison with the adaptive statistical iterative reconstruction technique. Eur Radiol..

[5] Korn A (2012). Iterative reconstruction in head CT: image quality of routine and low-dose protocols in comparison with standard filtered back-projection. Am J Neuroradiol.

[6] Love A (2013). Six iterative reconstruction algorithms in brain CT: a phantom study on image quality at different radiation dose levels. Br J Radiol.

[7] Oda S (2014). A knowledge-based iterative model reconstruction algorithm: can super-low-dose cardiac CT be applicable in clinical settings?. Acad Radiol..

[8] Hu-Wang E (2019). Chest CT Scan at Radiation Dose of a Posteroanterior and Lateral Chest Radiograph Series: A Proof of Principle in Lymphangioleiomyomatosis. Chest..

[9] Kim HJ (2016). Model-based iterative reconstruction in low-dose pediatric chest CT: comparison with adaptive statistical iterative reconstruction. Clin Imaging..

[10] Xu Y (2013). Impact of the adaptive statistical iterative reconstruction technique on image quality in low-dose CT. Clin Radiol..

[11] Miéville FA (2013). Model-based iterative reconstruction in pediatric chest CT: assessment of image quality in a prospective study of children with cystic fibrosis. Pediatr Radiol..

[12] Sun J (2017). Image quality improvement using model based iterative reconstruction in low dose chest CT for children with necrotizing pneumonia. BMC Medical Imaging.

[13] Sun J (2015). Improving pulmonary vessel image quality with a full model-based iterative reconstruction algorithm in 80kVp low-dose chest CT for pediatric patients aged 0-6 years. Acta Radiol..

[14] Sun J (2014). Image Quality in Children with Low-Radiation Chest CT Using Adaptive Statistical Iterative Reconstruction and Model-Based Iterative Reconstruction. PLoS One..

[15] Soysal N (2017). Non-invasive CT screening for pulmonary arteriovenous malformations in children with confirmed hereditary hemorrhagic telangiectasia: Results from two pediatric centers. Pediatr Pulmonol..

[16] Li HO (2015). High-pitch spiral CT with 3D reformation: an alternative choice for imaging vascular anomalies with affluent blood flow in the head and neck of infants and children. Br J Radiol.

[17] Hara AK (2009). Iterative reconstruction technique for reducing body radiation dose at CT: feasibility study. Am J Roentgenol.

[18] Leipsic J (2010). A prospective evaluation of dose reduction and image quality in chest CT using adaptive statistical iterative reconstruction. Am J Roentgenol.

[19] Vorona GA (2011). Reducing abdominal CT radiation dose with the adaptive statistical iterative reconstruction technique in children: a feasibility study. Pediatr Radiol..

[20] Prakash P (2010). Reducing abdominal CT radiation dose with adaptive statistical iterative reconstruction technique. Invest Radiol..

[21] ICRP (2013). ICRP publication 121: radiological protection in paediatric diagnostic and interventional radiology. Ann ICRP..

[22] Deak PD, Smal Y, Kalender WA (2010). Multisection CT protocols: sex- and age-specific conversion factors used to determine effective dose from dose-length product. Radiology..

